# Relationship between cardiac allograft vasculopathy and left ventricular diastolic dysfunction assessed by cardiac magnetic resonance imaging in heart transplant recipients

**DOI:** 10.1186/1532-429X-11-S1-P131

**Published:** 2009-01-28

**Authors:** Haruhiko Machida, Shinichi Nunoda, Kiyotaka Okajima, Kazunobu Shitakura, Akihiko Sekikawa, Yutaka Kubo, Kuniaki Otsuka, Masami Hirata, Shinya Kojima, Ai Masukawa, Satoru Morita, Kazufumi Suzuki, Mikihiko Fujimura, Eiko Ueno, Yoshiaki Komori

**Affiliations:** 1 Tokyo Women's Medical University Medical Center East, Tokyo, Japan; 2 Siemens-Asahi Medical Technologies Ltd., Tokyo, Japan

**Keywords:** Cardiac Magnetic Resonance, Cardiac Magnetic Resonance Imaging, Left Ventricular Diastolic Dysfunction, Heart Transplant Recipient, Cardiac Allograft Vasculopathy

## Introduction

Cardiac allograft vasculopathy (CAV), a major late complication that limits long-term survival of heart transplant recipients, is typically characterized as a diffuse concentric intimal hyperplasia of the coronary artery. Invasive coronary angiography/intracoronary ultrasound (ICUS) are widely performed for CAV screening of patients with no ischemic symptoms from their denervated hearts before congestive heart failure, cardiac arrhythmia, or sudden death occurs. This vasculopathy usually accelerates left ventricular diastolic dysfunction before systolic dysfunction. Cine images on cardiac magnetic resonance (CMR) examination, the most accurate test for cardiac functional analysis in the clinical setting, easily quantify peak filling rate (PFR) as an index of left ventricular diastolic function. This measurement can noninvasively predict early-stage CAV and be useful for risk stratification and adequate patient management. On a single comprehensive CMR examination, we can also assess asymptomatic systolic dysfunction of the left ventricle from cine images and myocardial infarction or scar formation from late gadolinium-enhanced (LGE) images.

## Purpose

We investigated the clinical feasibility of CMR imaging for noninvasively screening for CAV in asymptomatic recipients of heart transplants. Especially, we assessed the significance of left ventricular PFR value as a marker of early-stage CAV over several parameters of the systolic function and LGE by CMR imaging.

## Methods

Between June 2006 and June 2008, 38 asymptomatic recipients of heart transplants (25 men, 13 women, aged 37.2 ± 14.9 years) underwent both CMR and ICUS 8.5 ± 4.4 years after heart transplantation. We measured PFR normalized to end-diastolic volume and several parameters for systolic function of the left ventricle, including ejection fraction (EF), stroke volume (SV), and cardiac output (CO), by steady-state free precession cine CMR imaging with 20 sampling phases during one cardiac cycle. We also evaluated intramyocardial LGE on the CMR examinations. According to Stanford classification based on intimal wall morphology assessed by ICUS [1], we classified recipients of grade 0–2 as negative and grade 3–4 as positive for CAV and compared the values of PFR, EF, SV, and CO between the 2 groups using Mann-Whitney U test. *P* < 0.05 was considered statistically significant. Furthermore, we calculated receiver operating characteristic (ROC) curve in the relationship between PFR value and CAV as defined by ICUS.

## Results

Using ICUS, we classified 20 patients (53%) positive and 18 (47%) negative for CAV. There was no significant difference in the values for EF (58.1 ± 4.8% versus 58.8 ± 7.1%, *P* = 0.85); SV (43.0 ± 12.3 mL versus 49.4 ± 17.6 mL, *P* = 0.16); and CO (3.41 ± 0.81 L/min versus 3.83 ± 1.54 L/min, *P* = 0.46). No patient revealed intramyocardial LGE.

In contrast, the PFR value was significantly lower in the positive (3.63 ± 0.90 EDV/sec) than negative group (4.43 ± 0.84 EDV/sec, *P* = 0.01). Area under the ROC curve was 0.759 (95% confidence interval: 0.587 to 0.882). When PFR cut-off value was 3.65, CAV sensitivity was 58.2% and specificity, 79.8%. Table 1.

**Table 1:** Relationship between cardiac allograft vasculopathy and parameters for left ventricular function

	CAV positive	CAV negative	P value
PFR (EDV/sec)	3.63 ± 0.90	4.43 ± 0.84	0.01
EF (%)	58.1 ± 4.8	58.8 ± 7.1	0.85
SV (mL)	43.0 ± 12.3	49.4 ± 17.6	0.16
CO (L/min)	3.41 ± 0.81	3.83 ± 1.54	0.46

## Conclusion

The presence of CAV significantly enhances diastolic dysfunction of the left ventricle. For asymptomatic recipients of heart transplants, PFR measurement with CMR provides noninvasive prediction of CAV, which precedes systolic dysfunction and myocardial infarction or scar formation, and PFR is a feasible tool for decision-making in managing those patients.
